# Oral health problems linked to obstructive sleep apnea are not always recognized within dental care—As described by dental professionals

**DOI:** 10.1002/cre2.517

**Published:** 2021-11-17

**Authors:** Kristina Berggren, Anders Broström, Allen Firestone, Bridget Wright, Eva Josefsson, Ulrika Lindmark

**Affiliations:** ^1^ Center of Oral Health, School of Health and Welfare, Jönköping University Jönköping Sweden; ^2^ Department of Nursing School of Health and Welfare, Jönköping University Jönköping Sweden; ^3^ Department of Clinical Neurophysiology Linköping University Hospital Linköping Sweden; ^4^ Division of Orthodontics Ohio State University Columbus Ohio USA; ^5^ Division of Dental Hygiene Ohio State University Columbus Ohio USA; ^6^ Odontologiska Institutionen Department of Orthodontics Jönköping Sweden; ^7^ Department of Health Sciences Karlstad University Karlstad Sweden

**Keywords:** dental hygienist, dentist, health, obstructive sleep apnea, oral health

## Abstract

**Objectives:**

Obstructive sleep apnea (OSA) has an impact on an individual's quality of life and general health, and can also affect their oral health. The patient's experiences, together with intraoral signs and symptoms could indicate the presence of OSA. Knowledge that the patient has, or is at high risk for having OSA can help the dental healthcare provider maintain the oral health and general health for these patients. The purpose was to explore dentists and dental hygienists' experiences when encountering adult patients with potential, untreated and treated OSA.

**Methods:**

A qualitative inductive approach was used. Experienced dentists and dental hygienists working within Swedish Public Dental Service were strategically selected. Semi‐structured face‐to‐face interviews were performed followed by qualitative content analysis.

**Results:**

Interviews from 13 participants, seven dental hygienist and six dentists, led to three areas describing varied experience: Importance of the patient encounter and identifying intraoral signs both of which describe experiences related to the importance of the initial unstructured conversation and focused clinical assessments, and strategies for nurturing care which point to interest about care, treatment, and collaborations with medical health care providers.

**Conclusions:**

Dental professionals are not able to consistently recognize patients who have, or are at high risk for OSA. During the patient encounter, is it important to determine if a patient is at risk for, or has oral signs of OSA.

## 1 INTRODUCTION

Obstructive sleep apnea (OSA) is a multifaceted sleep‐related breathing disorder (Heinzer et al., 2015; Senaratna et al., 2017). Its association with hypertension, metabolic‐ and cardiovascular disease is well documented (Drager et al., 2010; Fu et al., 2017), but recently periodontal disease has been suggested to be associated with OSA (Ahmad et al., 2013; Vuorjoki‐Ranta et al., 2016). Common signs and symptoms during sleep are loud snoring and consider interruptions of breathing, such as apneas (i.e., total obstructions of the upper airway) and/or hypopneas (i.e., partial obstructions of the upper airway) despite continued respiratory movements (George & Ferraro, 2015). Restless sleep may cause several daytime symptoms such as morning headaches, listlessness, depressive symptoms, and cognitive dysfunction, but excessive daytime sleepiness is probably the most important one due to an increased risk for traffic and occupational accidents (Epstein et al., 2009). The severity of OSA is expressed as the average number of apneas and hypopneas per hour of sleep, i.e., the Apnea‐Hypopnea Index (AHI). Mild, moderate and severe OSA are defined as AHI 5‐14.9, 15‐29.9 or ≥30, respectively (Epstein et al., 2009). The prevalence of OSA in a general adult population has previously been described to be as high as 40% (Franklin & Lindberg, 2015; Senaratna et al., 2017), but due to its close association to obesity, OSA is likely to be an even larger future health problem (Franklin & Lindberg, 2015).

Treatment options for OSA include continuous positive airway pressure (CPAP), oral appliance therapy (OAT), behavioral modification (i.e., weight loss, positional therapy) or surgery (Ho & Brass, 2011; Kulkas et al., 2013). CPAP, predominantly used in cases with moderate and severe OSA, can reduce or abolish symptoms, improve quality of life, and reduce the risk of morbidity and mortality (Ward et al., 2014). A significant problem with CPAP is its poor long‐term adherence, which can be positively influenced by device‐related interventions, handling of side effects, as well as interventions strengthening treatment motivation (Bakker et al., 2019; Brostrom et al., 2018; Ward et al., 2014). OAT is another effective treatment alternative predominantly used in cases with mild or moderate OSA (Nordin et al., 2016). Side effect such as discomfort, jaw pain and bite changes are reported, and a positive outcome is also dependent on an adherent patient (Sheats et al., 2017). Untreated OSA, or patients who are non‐adherent to their treatment, suffer poorer quality of life and increased risk of morbidity and mortality (Silva et al., 2016). A common side effect of CPAP and OAT as well as untreated OSA is dry mouth (Nordin et al., 2016; Sheats et al., 2017).

There are several intraoral signs and symptoms associated with OSA that can be detected by dental professionals during routine clinical encounters (Intraoral signs include redness of the soft palate and uvula area, narrow palate, enlarged tongue and torus mandibularis [>2 cm on both sides of lower mandible] and symptoms include dry mouth, bruxism, retrusive jaw). Although these signs and symptoms are not necessarily caused by OSA, proper identification and understanding of the association with OSA by dental professionals, may help to recognize the need for further evaluation of OSA (Ruangsri et al., 2016; Vuorjoki‐Ranta et al., 2016). Simple clinical indices, for example, the Mallampati index, are used to predict the likelihood that a patient has or may develop OSA (Ruangsri et al., 2016). Short validated questionnaires, for example, the STOP‐Bang, can also be used by dental professionals in conjunction with the medical history when screening for OSA (Chung et al., 2008; Chung et al., 2012; Mallampati et al., 1985). OSA affects general health and oral health, therefore dental professionals must be seen as an important resource within health care for detecting OSA and identifying non‐adherent CPAP or OAT users in need of support.

Even where signs and symptoms indicate that a patient is at high risk for OSA referrals for diagnostic procedures and treatment follow‐up are commonly omitted (Marin‐Oto et al., 2019). As a result the majority of people with OSA are undiagnosed, and diagnosed patients lack clinical support with their treatment (Epstein et al., 2009). Because many patients in developed countries go for regular dental check ups so dental professionals could help identify individuals with potential OSA through the patient medical history and oral clinical signs and symptoms. Dental professionals can refer undiagnosed high risk patients to sleep practitioners and also identify those who are diagnosed but non‐adherent and support them with their treatment needs (Ahmad et al., 2013; Jauhar et al., 2010; Vuorjoki‐Ranta et al., 2016).

A few previous studies have indicated that experiences of clinical encounters between patients with OSA and dental professionals can vary depending on the professional's educational background, clinical experience, appointment setting and the patient's situation (An & Ranson, 2011; Jauhar et al., 2010; Reibel et al., 2019). Lack of knowledge or confidence about OSA can also contribute to the dentist's failure to diagnose or support patients undergoing treatment (Reibel et al., 2019). Meetings between dentists and patients with potential, untreated or treated OSA have rarely been described and the current knowledge gap provides scope for a study with exploratory design. Therefore, to gain a deeper understanding and capture the qualitative nuances of meeting patients with potential, untreated or treated OSA from a dental professional perspective, a qualitative study was conducted. The purpose of this study was to explore dentists' and dental hygienists' experiences when encountering adult patients with potential, untreated or treated OSA, via in‐deep interviews.

## 2 MATERIAL AND METHODS

A qualitative, inductive approach was used for data collection and analysis (Moon et al., 2013). Data collection occurred at the Public Dental Services in two counties in the southeast part of Sweden. Semi‐structured interviews were used to examine dentists and dental hygienists encounters with patients with potential, untreated or treated OSA. Content analysis, a common and well‐structured research method, was implemented to identify pattern in recorded communications (Graneheim & Lundman, 2004).

### 2.1 Participants and sampling

The head of the Public Dental Services in the two counties approved the study protocol and recommended dental clinics with potential participants according to the inclusion criteria. To achieve a nationally representative sample, eight large dental clinics were selected based on their location, size, and routines to be representative of dental care in Sweden. Inclusion criteria for the participants included registered dentists and dental hygienists who are employed by one of the eight selected clinics. Potential participants were then identified from a list and contacted by email.

### 2.2 Interview procedures

All interviews were conducted by one of the researchers (K.B.) from February to April 2019. A vignette technique has been shown to be a useful trigger resulting in rich descriptions in qualitative studies (Azman & Mahadhir, 2017). This technique was used with the intention of collecting a wide variety of experiences from the dental professionals. Three scenarios (Appendix A) describing patients with potential, untreated or treated OSA were created by the first author (K.B.) together with two experienced researchers/clinicians within oral health (U.L.) and OSA (A.B.). The scenarios, including both text and pictures, were based on the Mallampati index class III (soft palate and base of uvula visible, high risk for OSA) and class IV (only hard palate visible, high risk for OSA) (Mallampati et al., 1985). The investigators created an interview guide including open‐ended questions followed by probing questions to collect an in‐depth understanding of the practitioners understanding of encounter OSA patients at a dental clinic. Together with the scenarios, open‐ended questions were asked; “Can you tell me about your clinical experiences with patients with OSA?,” “What is your experience about OSA in relation to other diseases?” “How often do you meet similar patients?,” “Describe how you treat these patients,” “Describe what kind of information you give the patient.” Follow‐up questions adapted to the conversation were asked such as: “Can you describe this more?,” Can you develop what you mean?,” “Please, explain.” Three pilot interviews were performed to test the scenarios and interview guide. Despite showing good results, the pilot interviews were not included in the study. The interviews were performed in a place chosen by the participants either a quiet room or the coffee room at the clinic. Before the interview started, each participant was given a detailed description about the study and received time to read and reflect upon the three scenarios. Detailed field notes and a project diary were recorded throughout the data collection phase. By noting gestures and breaks during the interview, experiences of OSA, both reliability and credibility were insured. Interviews ranged in duration from 20 to 30 min and were recorded and transcribed verbatim by the first author (K.B.).

### 2.3 Data analysis

The data analysis was performed by content analysis as described by Graneheim & Lundman (2004). A full transcript of all interviews was read several times by the first author (K.B.) and the analysis process was performed in steps (Table 1). First, all transcripts were read to find meaning units in the text that related to the aim of the study. Based on the meaning units that were identified, the transcripts were coded and then categorized into subcategories and categories in a two‐step process. In the first step three members of the research team (K.B., A.B., U.L.) with extensive clinical experience in both oral health and OSA, as well as qualitative research, discussed data and developed a preliminary version of the categories. In the second step, three researchers (A.F., B.W., E.J.) with broad clinical experience in both oral health and OSA joined the discussion until a consensus agreement was reached regarding the analytical process.

**Table 1:** Example of the data analysis based on meaning units, codes, subcategories, and categories

Meaning units	Code	Subcategories	Categories
Dentist 1 …Contact healthcare…to do an investigation…maybe I ask how often they go to the doctor…how long ago it was…I can ask sometimes…eh…	Questions concerning general health connected to the oral health	Cooperation between dental care and health care Health history gives information about OSA	Strategies for nurturing care
Dental hygienist 3 …I ask about health and so on…but it is maybe not always there either comes up…but sometimes patient say that they have one CPAP or that they use an oral appliance therapy.	By health history Patients tells that they use CPAP or oral appliance therapy	Health history gives information about OSA	Importance of the patient encounter

### 2.4 Ethical considerations

The guidelines for medical research involving human subjects in the Declaration of Helsinki were followed (Ethical Declaration of Helsinki, 2008). An approval of the study was given by the local University after a careful evaluation of the study (i.e., including design, sampling, data collection procedure, presentation of the results and dissemination of the findings). All participants received written and oral information about the study and gave informed written consent. During the interviews, no sensitive personal data were collected. The questions were based on the experiences of providers meeting and examining patients with OSA in general and no specific information regarding a third party was asked for. The presentation of text in the results, and the selection of quotations was made with the intention to decrease the risk for identification of the participants.

## 3 RESULTS

### 3.1 Participants

Thirteen participants were interviewed, six dentists and seven dental hygienists, two males and eleven females. The age range of the participants was 21–62 years, with a median age of 44 years. The number of years of clinical experience varied (2–36 years). The years of the professional education varied, with dental hygienists having one to 3 years and dentist having 5 years of education.

### 3.2 Thematic analysis result

**Figure 1:**
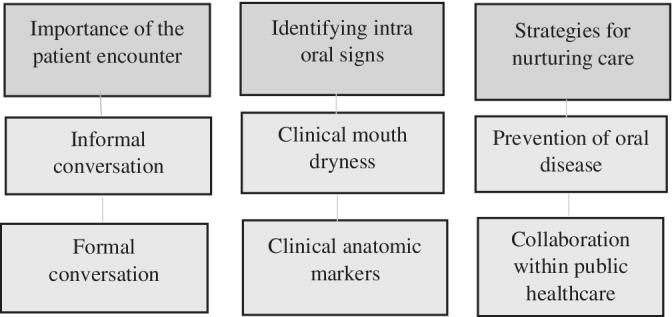
Overview of categories and subcategories

Three categories were created from the dentists' and dental hygienists' experience of encounter adult patients with potential, untreated or treated OSA. Two of the categories, importance of the patient encounter and identifying intraoral signs described situations, circumstances and clinical assessments, that could prompt the participants for a more in‐depth conversation about OSA. The third category, strategies for nurturing care, described experiences reflecting on care, treatment and collaborations. Categories and subcategories identified in the study are shown in Figure 1. The subcategories are further described below.

### 3.3 Importance of the patient encounter

The appointment with a patient contains both informal and formal conversations. Several participants described how the relationship with their patients was created through mutual trust and good relations, both by a social informal and formal conversations about OSA‐related issues. Both these conversations were valuable triggers for further questions about OSA‐related factors. However, participants reported a lack of discussion and ultimate care and treatment of OSA due to a lack of knowledge about OSA or the oral‐systemic link.

#### 3.3.1 Informal conversation

The informal conversation, often based on a long‐term relationship with the patient, can be described as a social approach which often could start in the waiting room or during the walk to the treatment room before the clinical examination began. During this informal conversation, information could emerge that their sleeping partner had noticed that they were snoring with breathing pauses.

“Just by a glance…already in the waiting room you make an assessment…. Many times, you have worked for such a long time that you have built a relationship…” (Dental hygienist 7)

“…it's most common that the patient asks about [OSA]…and then you take it from there…” (Dentist 3)

The patients seemed to initiate this informal conversation related to trouble with snoring, headache, and fatigue. According to the participants, it seemed that during these short conversations patients were forthcoming this type of information to the dentist/dental hygienist even before the professionals asked about it. Based on this type of informal conversation, several participants reported identifying patients who potentially had OSA or were being treated with OAT and/or CPAP. Some specific information participants noted from their personal experiences included a higher prevalence in men vs. women, weight loss could reduce the need for use of a CPAP, and patients often complain of daytime fatigue.

#### 3.3.2 Formal conversation

For many participants, the formal conversation based on questions from the medical history as well as their knowledge related to the oral‐systemic link, helped them to identify patients with potential OSA, as well as their awareness of the relationship between oral related conditions and general health.

“Sometimes I ask if the patient is snoring, I definitely do… It's often seen amongst overweight middle‐aged men…if you ask, you get a hundred times out of a hundred, a yes, on that question…” (Dentist 2)

For some participants, the formal medical questions became a natural part of a patient‐centered conversation with the patient. Through this conversation an understanding could be created on a more personal level to provide information regarding, for example, morning fatigue. This was especially noted, if there was information in the medical questionnaire that indicated that the patient was snoring or being treated with OAT or CPAP or questions related to occlusion, jaw joints, or headaches.

“They often raise it [OSA] themselves…but there is a question in the medical history that can report it…snoring… Yes…” (Dental hygienist 6)

Some participants omitted ignoring questions or symptoms from patients related to OSA. This could be related to the fact that the medical history did not include that kind of question, that another treatment was planned, or lack of time or lack of knowledge about the relationship between OSA and oral health.

“I don't know if our way to collect data regarding medical history is to trust…the questions that are put there are not focused on it [OSA]…” (Dentist 3)

Some participants reported their previous experience with patients who are being treated with OSA also had general health problems such as diabetes, cardiovascular disease, obesity, and were older. Their previous experience enabled them to be able to identify other patients with potential OSA while they are reviewing their medical histories. Participants who did not have these previous experiences were unable to associate various diseases with OSA. When asked about the association between certain medical conditions and OSA, one participant reported.

“I'm very, very unsure what can cause the snoring… Yes… I don't know if it will appear in the medical history… Alcohol consumption, obesity and so on, but it's just as well that I say that I am very poor at different kinds of poor health conditions…” (Dentist 3)

### 3.4 Identifying intraoral signs

Another important part of the appointment with patients included oral clinical examination and treatments. This category also describes the concern about not identifying important clinical oral signs and how to handle the information if signs were found. The category identifying intraoral signs encompassed the clinical markers clinical mouth dryness and clinical anatomic markers. Some participants reported having clinical experiences with different intraoral signs associated with OSA. Sometimes the participants recognized the relationship, but other times there was no association made. If a patient had CPAP or OAT, the inspection of oral signs related to OSA was described as more obvious.

#### 3.4.1 Clinical mouth dryness

Dry mouth was noted in patients with OSA as both subjective symptom. The patient perspective, and objective. The professional perspective, a sign. Intraoral signs of dry mouth could be recognized by dry lips and tongue. When patients complain about dryness at night, participants were not able to associate these symptoms with snoring or OSA. Some participants were able to make a connection between CPAP use and dry mouth due to their knowledge of the device.

“Among other things, they have a mask [CPAP] at home and felt that they got a dry mouth…” (Dental hygienist 2)

Some of the participants did not associate oral dryness with OSA or its treatment with CPAP. Instead, they thought that the dry mouth could be due to other conditions, for example being old or a polypharmacy treatment.

“I associate dry mouth with the medication but not with apneas… at least I don't think so…” (Dental hygienist 3)

#### 3.4.2 Clinical anatomic markers

Different clinical anatomic markers were stated as possible links to OSA. Anatomical markers such as a broad, large tongue and a high, narrow palate were examples of such markers. But when anatomic deviations were found, they were not always recognized as a sign or feature related to OSA. During the examination and dental treatment, the participants expressed that they might note anatomic markers which were related to OSA. These notations were usually made to a large tongue and a high, narrow palate making it difficult when exposing radiographs or treating posterior teeth not because they are associated with OSA. Typically, this was documented as a “troublesome tongue.” Other structures described were reduced activity in the soft palate. Some of the participants had encountered patients who had a surgical reduction of the soft palate as a treatment for OSA. This procedure might also result in side effects like difficulty in swallowing. Even if oral anatomic deviations such as broad tongue and snoring were found, they were not always linked to signs of OSA. A big tongue, as well as a mouth with narrow high palate could be linked with the patient snoring, but they were also likely to be linked to other different treatment problems, such as some prosthesis therapy.

“It's often you see some “life,” an extra liveliness in the soft palate… you notice a difference compared to other patient categories. It's [the soft palate] kind of flaccid and does not have that tension really…you can then see that they get to work a little with the muscles at the back of the tongue and such…” (Dentist 1)

“When I look at a palate, I usually think more about whether or not a full denture could be done…” (Dentist 3)

Some participants did not observe the palate at all.

“I don't look at the palate… no I don't…” (Dental hygienist 6)

Participants noted that with those patients using OAT, bite marks in the oral mucosa was a common sign, where mesial occlusion (class III), were more prominent. From that aspect, the participants felt more secure with patients treated with CPAP than those who used the OAT.

“When I have such a patient… I usually check the bite a little bit, to see if it changes… it can do that in some patients…” (Dentist 2)

Although, some clinical anatomic markers were described and documented in the patient's record, participants reported that they were unsure if there was any follow‐up beyond the documentation. Participants also reported that they may have missed the clinical anatomic signs and/or the OSA symptoms that were being reported by the patients.

“But I think we miss a lot… it is clear if someone has not felt healthy in a few years and has a hard time getting up in the morning…likewise if she arrives breathless… I think it's noted, but that you do not proceed with it…” (Dentist 3)

### 3.5 Strategies for nurturing care

The category strategies for nurturing care included the subcategories prevention of oral disease and collaboration with public healthcare. This category was triggered with the vignette technique with patient cases and the participants' thoughts and experiences of encountering these patients in a clinical situation. The participants recognized at least two of the three cases, presented that is, the fictitious patient cases describing potential and treated OSA. The vignette technique gave the participants a sense of realistic situations, which they could recognize, from their everyday clinical experiences. The subcategories describe two perspectives of strategies for nurturing care. First, the participants described thoughts and experiences about preventive treatment for oral health for patient with OSA or related symptoms, that is, snoring. Second, oral treatment in relation to general health and also in relationship to collaboration with the public healthcare service.

#### 3.5.1 Prevention of oral disease

Preventing oral disease emerged from the participants' experience of how to best treat patients with OSA. However, prevention had more of its origin from snoring rather than the link to OSA. The participants focused mostly on the dry mouth rather than the cause of snoring. The attention was on information, as well as supportive care with the aim of prevention. Most of the participants voiced responsibility for providing the patient with information that snoring can cause dry mouth. In addition to information about dry mouth, most of the participants recommended fluoride rinses with 0.2% NaF and tablets preventing oral dryness. Patients' self‐care of their oral hygiene was also expressed as important.

“I usually stress how important it is to take extra fluoride and give information about different products that are available… I hand out some brochures and information sheets about it… It's clear that you have to be extra careful with oral hygiene when you have dry mouth, it's a part of the cause…” (Dental hygienist 3)

Dry mouth was also associated with oral diseases like caries. Some participants assigned preventive measures to a colleague at the clinic to deliver more caries preventive information depending on the dry mouth.

“Dry mouth can be a problem… if I don't have the time [to give information]… the patient can meet our dental hygienist…” (Dentist 1)

#### 3.5.2 Collaboration within public healthcare

The collaboration between dental‐, and other healthcare services was perceived by most of the participants as positive and essential. According to the participants, the general health view was based on a “whole person concept” where dental care was an important part of the healthcare chain, which could help patients with OSA more comprehensively. Most of the participants expressed the feeling that dental care could be better if the dental professionals saw the patient from a more holistic perspective, not just focusing on oral diseases.

“Absolutely, it is very important that you have this cooperation with the healthcare, see the whole body… “the mouth belongs to the body”… so you should get help…it is important, I think…” (Dentist 4)

Thoughts about collaborations with general healthcare were positive, mainly based on avoiding the responsibility for caring for patients with signs of OSA. These thoughts were similar to those related to other general diseases and their relation to oral health. A common planned measure was to consult a colleague at the clinic or recommend the patient seek general healthcare.

“Absolutely it [collaboration] would be great…however, taking responsibility for it [OSA]…is another matter…” (Dentist 1)

Collaboration in other areas of general and oral health was described as working well. However, receiving more knowledge about the symptoms and intraoral signs related to OSA was viewed as desirable. Also, to facilitate collaboration with the health service, formal structures or strategies about how to take care of these patients were seen to be important.

“Yes, it [OSA] really is a disease like any other… so we should have much more knowledge. If you have knowledge and know what you're talking about, it becomes more natural to link it to questions that already exist… after all, we usually have a decent collaboration. So absolutely, why shouldn't we have a collaboration on this as well…” (Dental hygienist 4)

Most of the participants were aware of how OSA was being handled in general public healthcare. The majority were aware that the examination was made by general health practitioners who then referred patients to dental care to deliver OAT. Some participants also expressed knowledge of the referral process in conjunction with CPAP treatment from the general health practitioner to a specialist in ear nose and throat (ENT) care. Also, the healthcare recommendations to report diagnostic results with the AHI was mentioned by some participants. However, the participants were less experienced in determining the appropriate treatment option (i.e., CPAP or OAT).

“I understand that they are being explore and that they get to sleep, and that something is measured…those I have met, say in passing that you get to sleep and that they measure how much…yes that's what I know about it… what level that determines whether you get a machine [CPAP] or not… I do not know…” (Dental hygienist 1)

Participants were concerned about the lack of knowledge about the relationship between oral health and OSA. They suggested that there should be collaboration between the general healthcare provider and the dental professional during the management and treatment of OSA.

## 4 DISCUSSION

The purpose of this study was to explore experiences of encountering adult patients with potential, untreated or treated OSA. The results showed that dental professionals' experiences with OSA varied and that possible oral health conditions as a result of OSA, were not always recognized. These results are in agreement with other studies (Bian, 2004; Jauhar et al., 2010; Reibel et al., 2019), showing that lack of knowledge among dentists and dental hygienists about OSA and its treatment may result in difficulties detecting the condition and related oral health problems. A main theme from the participants in the present study was the need for more knowledge about OSA and a request for tools to identify patients with potential, untreated or treated OSA. Participants also identified the need for collaboration with general healthcare providers who would have the main responsibility for treating patients with OSA.

Analyzing the interviews resulted in three main categories: Importance in the patient encounter, identifying intraoral signs and strategies for nurturing care. The overall interpretation of the results is that the dental professional's experience of encountering patients with potential, untreated or treated OSA is derived from two parts. First, any conversation about OSA is based on a long, trusting relationship between the patient and the professional, built on the patient's history, values and preferences. Both the patient and the professional have an active role in the conversation, which is based on an awareness of the relationship between oral health and OSA‐related factors. Previous research has shown that communication is a “two‐way exchange,” with a foundation in evidence‐based care and an important part acknowledging and enlisting patient autonomy (Bae, 2017; Glick, 2019). Second, OSA is a disease that can lead to short‐term as well as long‐term physical, cognitive and emotional consequences if untreated (Marin‐Oto et al., 2019). Short‐term consequences of OSA include sleepiness, impairment of neurocognitive function and daytime performance. Long‐term, untreated OSA increases the risk for cardiovascular disease, cerebrovascular and metabolic syndrome disorders resulting in premature death (Marin‐Oto et al., 2019). In the current study, dentists' and dental hygienists' understanding of short‐ and long‐term consequences of OSA was expressed in the three categories that described their experiences of encountering patients with OSA.

Importance in the patient encounter, the first category, showed that experiences gained in informal conversation created both trust and knowledge for the patient and the participants and presented an opportunity to identify short‐term consequences of OSA. The participants also described the importance of obtaining awareness, knowledge, and responsibility from the patient in the informal conversation regarding OSA. Frankle & Stein (Frankel & Stein, 2001), have explained the informal and the formal encounters as occasions to build trust, and comfort between the patient and the health care provider and as an opportunity for an effective delivery of information. They present these encountering as a socialization between the patient and the participants, which create positive health outcomes. A trustful relationship leading to respectful conversations has been described as very important both within dentistry (Stenman et al., 2010) and general health care, involving both understanding and competence to establish a caring and trustful relationship (Berg & Danielson, 2007). We found that during the formal conversation (i.e., about the patient's medical history) some of the dentists and hygienists described learning about long‐term complications of OSA related to general health (e.g., diabetes and cardiovascular disease). This underscores the importance of the patient perspective in the verbal part of the encounters and the professional's responsibility to bring that information into the clinical examination to identify intraoral signs of OSA or subsequent dental problems (An & Ranson, 2011).

Intraoral signs, the second category, was most frequently about oral dryness, but several clinical anatomic abnormalities were also mentioned. However, the abnormalities were not always expressed as a problem from the patient perspective, for example an overactive, broad tongue. The patient perspective of OSA and oral health was not investigated in this study, but it is important that future studies address this area. An uncertainty about OSA and its relation to oral health was expressed by the professionals, and they frequently expressed a wish for valid and reliable indexes to detect OSA. This is in agreement with the results of other studies (Bian, 2004; Reibel et al., 2019).

Early treatment can have a beneficial impact on a patient's health and quality of life (Kale et al., 2018) and, there are some tools to anatomic conditions and symptoms that suggest patients are at high risk for OSA. Screening indexes, for example, the Mallampati classification (Ruangsri et al., 2016), the STOP‐, and the STOP‐Bang questionnaire, can provide an opportunity for dentists and dental hygienists to identify patients at risk for OSA (Kornegay & Brame, 2015). Screening methods, like the Mallampati index and STOP‐Bang questionnaire are effective methods in general healthcare, which can also be used by dental care professionals (Ho & Brass, 2011). CPAP and OAT can effectively treat OSA provided that the patients use it regularly. However, patient adherence has been shown to be a significant problem (Nordin et al., 2016; Yetkin et al., 2008). It is therefore of great importance that patients also have professional support for their treatment. Dentists and dental hygienists could be that support, as patients often meet dental professionals on a regular basis.

Strategies for nurturing care, the third category, was described as essential by the participants. This category encompassed both strategies for oral care within the dentistry, as well as the importance of collaborating with practitioners from general healthcare. The choice of oral care for individuals with OSA that the dentists and dental hygienists provided was mainly based on oral dryness, but in most cases without any clear connection to OSA. However, oral dryness is a common overall consequence for many diseases and medications, not only among patients with OSA (Oksenberg et al., 2006). Based on oral dryness, the professionals perceived that the patients received good oral care advice, even if the association to OSA was not part of the discussion. Other documented oral signs related to OSA contributed to follow‐up questions, for example about breathing events, such as apneas, during sleep. Dental professionals have an interest in contributing to general health as well as to good oral health and, with knowledge about OSA they can recognize and properly interpret intraoral signs, such as abnormal anatomic structures (Kale et al., 2018). This category also included descriptions of collaborations with general healthcare professionals, where limitation of knowledge about OSA may be a barrier. These findings are in agreement with an earlier study (Bian, 2004). Being part of an interprofessional team contributes to collaboration where there is a reciprocity between different professions (Berlin & Sandberg, 2016). Within interprofessional teams, it is important that the result is close communication, mutual consultation and consideration for the other members (Thylefors, 2013). The target of interprofessional collaboration between dental care and general healthcare is to identify oral health, prevent disease and deliver high quality care to achieve improved health outcomes (Dolce, 2014). From this perspective, how to care for patients with potential, untreated or treated OSA must be improved.

Reflecting on the overall result, it is important to increase the knowledge about the associations between oral health and OSA. OSA must be seen as an oral health determinant that needs to be recognized in the dental care appointment and a natural part in oral clinical examinations (Jauhar et al., 2010). Education about this relationship should also support interprofessional collaborations, promoting quality care for patients with potential, untreated or treated OSA. This is also in line with the new oral health definition (Glick et al., 2016), including an extended holistic view of different domains that are essential for oral health. One domain of oral health, driving determinants, are factors that affect oral health: genetic and biological factors, social environment, physical environment, health behaviors, and access to care. This new definition and framework is used “to explain the multidimensions of oral health to our patients, other healthcare professionals, policy makers, and those others we seek to collaborate with and inform” (Glick et al., 2016). Thus, understanding associations between oral health and OSA and, interprofessional collaborations within healthcare is of importance. Our results show that more knowledge about OSA is required for dentists and dental hygienists. This additional knowledge along with validated tools to screen patients for OSA are important steps that would form the basis for improved patient communication and treatment within dentistry. In addition, this would support a more robust collaboration with public healthcare professionals.

### 4.1 Methodological reflections

Methods for measurement with focus on data sampling, analysis and results in the current qualitative study were performed according to established procedures to ensure high quality results (Graneheim & Lundman, 2004). The information was collected by interviews and analyzed by content analysis (Graneheim & Lundman, 2004; Moon et al., 2013) with the goal to gain deeper insight and describe dentists' and dental hygienists' experiences encountering OSA patients within dentistry. One of the strengths of the present study was the use of the vignette technique (Azman & Mahadhir, 2017). The vignette technique helped the participants to reflect and remember real patient situations and with the use of the Mallampati index the scenarios became a good icebreaker during the interview. The scenarios' description of possible patients guided the participants to view clinical situations even if they did not always have in‐depth knowledge about OSA. The participants were recruited from eight dental clinics of different sizes, they represented a wide range of ages and work experiences, which gave a varied and broad perspective. One limitation of the study was that the participants were not balanced according to sex, with females overrepresented. Another aspects that might be of importance is that some argue that research with qualitative design and small sample sizes are difficult to generelize. However, the results of this study will be valuable in designing further studies and educational interventions related to OSA and dental professionals in collaboration with general healthcare.

## 5 CONCLUSIONS

Dental professionals' in general dentistry experience with OSA varies and possible oral health problems like OSA are not always recognized. This was due to a lack of knowledge about OSA and of validated indices that could be used to detect risk for OSA. Knowledge about OSA as an oral health determinant is recommended and may promote interprofessional collaborations between dentistry and general healthcare.

## 6 CLINICAL RELEVANCE

### 6.1 Principal findings

In patient encounters, a two‐way communication about OSA and tools to detect oral health signs may increase dental professionals' ability to support patients with OSA. Collaboration between dental and public healthcare is essential for these patients.

### 6.2 Scientific rationale for study

The experience of the relationship between oral health and patients with potential, untreated or treated OSA is limited among dentists and dental hygienists. Thus, the area needs to be explored by an inductive approach to find solutions to increase qualitative and collaborative care for these patients.

### 6.3 Practical implications

More education about OSA to dental professionals, good communication skills and validated tools are important to support OSA patients.

## CONFLICT OF INTEREST

The author declare that they have no conflict of interest.

## AUTHOR CONTRIBUTION

**K. Berggren**, **A. Broström**, and **U. Lindmark** designed and planned for the study. **K. Berggren** made all the interviews and together with **A. Broström** and **U. Lindmark** conducted the primary analysis of the data. **K. Berggren**, **A. Broström**, and **U. Lindmark** wrote the paper. All authors contributed to the interpretation of the findings and reviewing drafts of the paper. The manuscript has been read and approved by all authors and each author believes that the manuscript represents honest work.

## Figures and Tables

**Figure A1:**
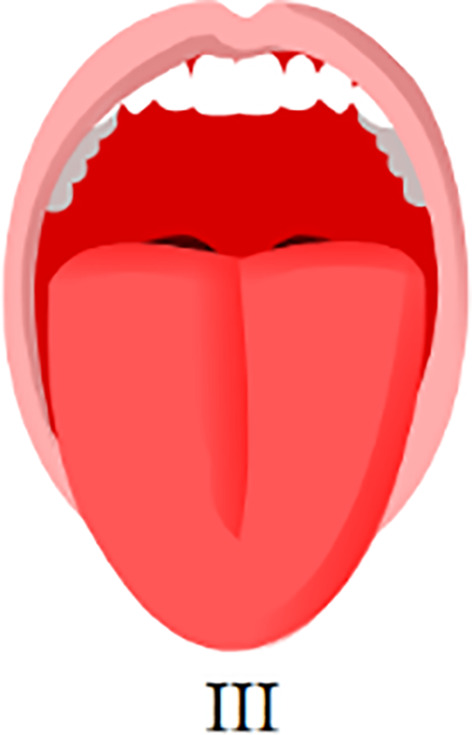
Modified Mallampati index III, (from Wikimedia Commons, the free media repository)

**Figure A2:**
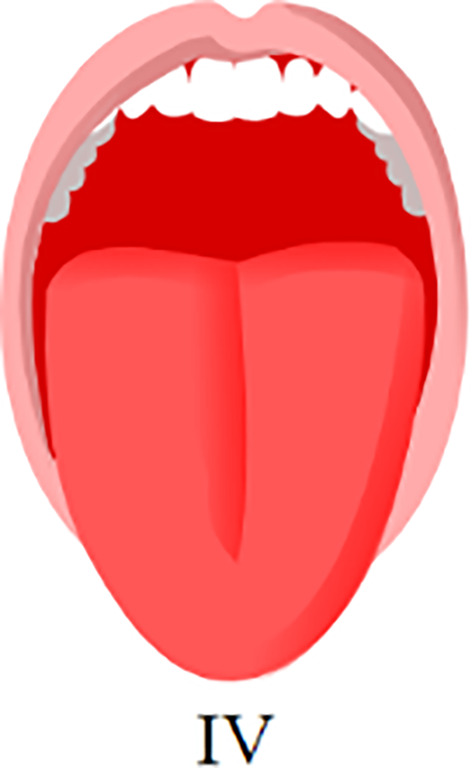
Modified Mallampati index IV, (from Wikimedia Commons, the free media repository)

## Appendix A DESCRIPTION OF THREE FICTITIOUS SCENARIOS USED DURING THE INTERVIEWS

### A.1. Ingress

To introduce and describe patients with potential, untreated or treated OSA with an intention of collecting a wide variety of experiences, fictitious scenarios were used. Before the interview started, the dental professionals were presented three scenarios, including text and pictures, based on the Mallampati index class III and class IV. The purpose of the scenarios was to give the participants a view of OSA‐related symptoms in different clinical patient situations even if they may not have comprehensive knowledge about OSA.

### A.2. Scenario 1: Patient with suspected but not diagnosed OSA

#### A.2.1. History

Your next patient is Marita. Today is the first time you will meet her.

Marita is 60 years old. She is short and clearly overweight. You notice that she is breathing heavily after the short walk from the sofa in the waiting room to the treatment room.

Marita has been married for 35 years; She retired 1 year ago due to her work environment becoming too physically demanding.

In the medical history, she states that she was diagnosed with high blood pressure and a clot in her right eye 8 and 3 years ago respectively. She has two prescription medications for hypertension and anticoagulant. She cannot remember the name of the medications. She often feels tired and the need to a “take a nap” in the afternoon.

You ask her if she smokes and she reacts strongly and bursts out:

- Smoking is the worst. I have never smoked. However, Marita mention she suffers from dry mouth, especially at night, and she also mentions that;
- My husband declares that I snore a lot. He has also experienced that I sometimes even stop breathing, he wants me to see a doctor.

Her oral hygiene regiment includes brushing twice per day with fluoride toothpaste and uses an interdental brush once per day. Marita sees the value in her oral health and seeks routine dental care once per year.

#### A.2.2. Clinical examination

The clinical intraoral examination shows objective dry mouth, brittle mucous membranes. The soft palate is visible, and the base of the uvula is seen (Figure A1).

### A.3. Scenario 2: Patient with untreated OSA

#### A.3.1. History

Peter, a 73 year old male, calls the dental clinic to cancel his appointment once again.

- “It's difficult for me to make it to the examination today.”

He says he prefers an afternoon time 2 months later.

- “Its difficult getting up early in the morning,” he says a little cautiously. “I'm not feeling well right now.”

This is his fourth cancelation and you feel a little hopeless, why does he cancel his appointment so often?

When you enter his record and read the last entry, it says Peter has not felt well in many years. There is no diagnosis in his medical history, and he is only taking one medication for asthma.

One week later, Peter calls the clinic urgently stating he has fractured tooth 46 which has damaged the lingua and he is in pain. When you arrive the treatment room, Peter is sitting in the treatment chair, but he is difficult to recognize due to gaining 30 kg + since the last time you saw him.

He talks about his dental problems and you notice that he is grateful that you are listening to him. When you ask him about his health, he mentions that he is very tired:

- “I can fall asleep anywhere and at any time,” he says, “and I also have problems with headache, especially early in the morning.”

He also mentioned that he is concerned about his dry mouth, that he is been smoking for 40 years and that he associates his smoking with this problem.

#### A.3.2. Clinical examination

During the intraoral examination you see a highly narrow palate and the lingua are broad and cover the tooth surfaces and the entire soft palate (Figure A2). On Peter's right side of the lingua you observe a fibrin‐covered sore with size of 10x10mm. You take an intraoral photo and polish the sharp edges of the tooth. A follow‐up appointment is scheduled to evaluate this area in 7 days.

### A.4. Scenario 3: Patient undergoing treatment for OSA

#### A.4.1. History

Bengt is late to the treatment as usual. He is always stressed and comes into the clinic out of breath. Bengt is 64 years old and works as a salesman at a medium‐sized company and he travels a lot for work.

- “Everything is just fine,” Bengt shrugs when you ask him the usual medical history questions.
- “The only “new” is that I got one of…what is it called again…” Bengt thinks a little before he bursts out—“a CPAP, I should wear it every night but that is difficult. Especially when I travel for work, I just forget to use it, or I pull it off during my sleep. I feel dryness in my mouth, it is like sand paper.” Bengt continues, “I'm not sure the CPAP works either.”

You know Bengt has been diagnosed with angina pectoris, hyperlipidemia, hypertension, and depression. The medications he uses is: Metoprolol, Thrombyl, Simvastatin, Propovan, Citalopram, Mirtzapine, Voltaren, Sandimun.

- “My dry mouth Continues to get worse says Bengt.
- “Do you have any samples for tablets to prevent the dry mouth?,” he asks happily.

#### A.4.2. Clinical examination

You will get started to treat Bengt and put the treatment chair to the right position.

- “Stop, do not put the chair down then I'll fall asleep,” Bengt bursts out.

In the clinical intraoral examination, you notice dry mouth and a large broad lingua that folds over the pharynx (Figure A2). During the treatment Bengt falls asleep and begins to snore.
